# Music Therapy with Preterm Infants During Kangaroo Care: A Mixed-Methods Feasibility Study on Physiological and Electroencephalographic Parameters and Parental Perspectives

**DOI:** 10.3390/children12030334

**Published:** 2025-03-07

**Authors:** Anna Carina Kriechbaum, Bernhard Csillag, Claudia Wenzel, Friederike Barbara Haslbeck

**Affiliations:** 1 Clinic of Neonatology, Kepler University Hospital (KUK), Level 4, 4020 Linz, Austria; bernhard.csillag@kepleruniklinikum.at; 2 Department of Medicine, Clinical Division of Palliative Medicine, Vienna General Hospital, Medical University of Vienna, 1090 Vienna, Austria; claudia.wenzel@meduniwien.ac.at; 3 Department of Neonatology, University Hospital Zurich, University of Zurich, 8091 Zurich, Switzerland; friederike.haslbeck@usz.ch

**Keywords:** music therapy, premature infants, neonatology, developmental support, EEG, skin-to-skin care, attachment, mixed methods

## Abstract

**Background:** Music therapy in neonatal care is a rising interdisciplinary interest in clinical practice and research. Studies showed that music therapy benefits preterm infants and their parents. We aimed to explore the possible influence of music therapy on physiological parameters in premature infants during skin-to-skin care and to assess the parents’ perspectives on music therapy and participation in the research project. **Methods:** The feasibility project was conducted in an Austrian neonatal intensive care unit with a mixed-methods design. The subjects were six preterm infants and their parents. We collected quantitative data on heart rate, oxygen saturation, and brain activity (EEG) and analyzed them descriptively. Qualitative interviews were conducted to explore the parents’ perspectives on music therapy and study participation and analyzed by using grounded theory coding. **Results:** The results of the quantitative data indicated a stabilizing effect on the vital parameters in the included premature infants. EEGs showed interburst intervals were longer during music therapy than before its application. Parents reported a great interest in the research project, an experience of deep relaxation through music therapy, and an intensification of their relationship with their infants. **Discussion/Conclusions:** Our feasibility sample indicates positive changes in the vital parameters and brain activity tendencies. The parents reported positive experiences and observations in their infants related to music therapy, and they enjoyed participating in the research project. However, the small number of subjects means that our descriptions should be interpreted cautiously, and more extensive investigations into EEG measurements in preterm infants are needed.

## 1. Introduction

According to Statistics Austria, 7.4% of all live births are premature, corresponding to approximately every 13th child in Austria [1].

Thanks to constant medical progress, premature babies survive from increasingly early gestational ages. This means that they must spend several weeks to months in a neonatal intensive care unit [2]. Preterm infants are functionally and structurally immature, and the premature transition from intrauterine to extrauterine challenges the entire organ system. The massive environmental change after birth can impact a premature baby’s respiration, circulation, nutrition, digestion, and heat balance, necessitating intensive medical care in most cases [3]. The babies are exposed to unknown stimuli, such as bright light, machine noises, and the unmuffled background noise of an intensive care unit, which they cannot yet sufficiently absorb and process [4].

Premature birth is traumatic for both infants and their families. “Premature” parents are ill-prepared for this new situation, which places them under considerable emotional strain that can affect their mental health [5]. The uncertainty of the baby’s health is a great burden, and the new situation triggers psychosocial stress [6].

Thus, premature birth presents parents, infants, and medical staff with many complex challenges, including neonatal environmental challenges. To cope with these, family-centered, needs-oriented care for premature babies and their parents is needed [7,8,9]. Music therapy is one family-centered approach to address these various and mutually interacting challenges with various approaches [10,11].

Creative music therapy (CMT) in the field of neonatology is described as a family-integrated early intervention to promote the development of premature babies through regulative, relaxing, and stimulating effects, strengthening the well-being of the parents and thus supporting the interaction and bonding process between parents and children [11]. In this process, the voice is used as gentle humming or singing in an improvised lullaby style. It is individually adapted to the breathing rhythm, the stage of development, various subtle reactions, and the needs of premature infants [11].

The positive effects of CMT on the development of premature infants, and specifically on functional brain activity and brain development, have been reported in a randomized controlled pilot trial demonstrating significantly improved functional processes between the thalamus and the cerebral cortex, stronger functional networks, and an improvement in the interaction of brain areas responsible for socio-emotional, cognitive, and motor functions [8].

A randomized controlled study by Giordano et al. [12] showed a positive effect of music therapy on the brain activity of preterm infants using amplitude-integrated EEGs. Additionally, a randomized controlled pilot trial by Olischar et al. [13] demonstrates a trend in the music intervention group towards a more mature sleep–wake rhythm using an amplitude-integrated EEG, suggesting a small beneficial effect of music on quiet sleep in neonates.

In a meta-analysis, significant positive effects of music therapy on the respiratory rate of preterm infants and maternal anxiety were demonstrated [14]. Yue et al. [15], who included 13 studies with 1093 subjects in their meta-analysis, showed a significant effect of music therapy on respiratory rate, heart rate, oral feeding volume, stress level, and maternal anxiety but not on oxygen saturation and behavioral status of preterm infants. Another interesting study demonstrates that music therapy effectively stabilizes preterm infants’ vital parameters (heart rate, respiratory rate, oxygen saturation), even when they are asleep. Therefore, music therapy can be considered an important non-pharmacological intervention in neonatal intensive care [16]. Similarly, mixed-methods studies demonstrate that music therapy positively affects the development of infants, parent–child bonding, parental anxiety, and stress levels [8,15,16,17]. The results of a qualitative study [18] illustrate the positive and formative impact of CMT on parents and their preterm infants through positive changes in parents’ early, nurturing, and musical interactions with their children, capacity building, and positive reinforcement.

Nevertheless, only a few studies have investigated the effect of family-centered music therapy on brain activity and development and have included the parents’ perspectives in their research. This mixed-methods feasibility study, therefore, aimed to evaluate the impact of music therapy during skin-to-skin care on the physiological parameters of preterm infants in a neonatal unit, particularly potential changes in brain activity (EEG), oxygen saturation, and heart rate before, during, and after music therapy. Additionally, this study aimed to evaluate how the parents felt about participating in a research project and what they experienced and observed regarding themselves and their premature babies during and after music therapy sessions.

## 2. Materials and Methods

### 2.1. Overview

This project was conducted as a feasibility study.

A mixed-methods convergent design was chosen to gain deeper insight into family-centered live music therapy in neonatology. This study investigated whether and how music therapy during KC influenced premature infants’ physiological parameters. Quantitative physiological outcomes included heart rate, oxygen saturation, and infant brain activity measured with electroencephalography (EEG) measurements. The parents’ subjective experiences of participating in this study and the music therapy were assessed through qualitative interviews and analyzed using grounded theory coding [19]. All parents were thoroughly informed about this study and confirmed their participation by signing the informed consent form.

This study was approved by the Ethics Committee of Upper Austria, application no: 1245/2019.

### 2.2. Setting

Data were collected in one session per subject between May and July 2020 at the Clinic for Neonatology, Kepler University Hospital (KUK), Level IV. Data collection was conducted in the patient rooms of the neonatal intensive care unit (NICU), mostly with fellow patients present. Music therapy has been part of standard care at this unit since February 2019. Quantitative data collection took place during KC (skin-to-skin contact between one parent and their child), which is provided by parents as standard care in the NICU.

### 2.3. Subjects

Preterm infants were eligible for inclusion if they were born between 29 + 0 and 33 + 6 weeks of gestation and were subsequently cared for in the NICU. The prerequisites for inclusion were the stability of certain physiological parameters in the infants (e.g., not intubated and with only moderate oxygen support) and an expected hospitalization until data collection was completed. Exclusion criteria included deafness, brain hemorrhages, skin diseases, syndromal or neurological disorders, unstable physical conditions, or transfer to another hospital. Additionally, eligibility for kangaroo care was assessed by the physician. If ongoing clinical deterioration was observed, such as increasing oxygen requirements, hypotension, or other signs of decline, kangaroo care was not permitted, and the patient was excluded from this study.

Parents were included if they had a stable state of health (no diagnosed mental and physical health issues), sufficient knowledge of the German language for data collection procedures, a minimum age of 18 years, physical presence/attendance for data collection during KC, and their written informed consent.

Participants were selected by the principal investigator and the second author.

### 2.4. Data Acquisition

#### 2.4.1. Quantitative Data

Data were collected once for each patient, one to two weeks postpartum, to avoid adding unnecessary stress immediately following delivery.

All subjects were exposed to music therapy at least once before data collection started. The research team integrated data collection into the regular ward routine and conducted it during standard KC to avoid creating an artificial situation and a possible bias in the data. All participants were given caffeine to activate the respiratory center, except for subject No. 5. Music therapy was primarily provided by nursing staff after routine care activities and occasionally before or after feeding. The infant had enough time to recover from the placement of EEG electrodes.

The quantitative data collection consisted of measurements of heart rate, oxygen saturation, and brain activity during KC. The values of the standard Philips IntelliVue X3/MX100 monitor were used for the quantitative data collection of oxygen saturation and heart rate.

Before music therapy, the preterm infants were connected to EEG electrodes for data collection to measure brain activity before, during, and after music therapy. Subjects and a parent could be observed by a video camera during data collection. An EEG (Natus neurology) with a neo circuit consisting of 8 channels and 11 electrodes was used by an EEG team from the university hospital, attaching gold cup adhesive electrodes to the infants’ heads using an adhesive paste. The principal investigator (AK), the senior physician (BC), and the EEG team positioned the EEG device (Natus neurology) video camera to observe the reactions of parents and their preterm infants during data collection. The camera (Sony HD) was used exclusively to support the quantitative analysis. Afterward, a nurse transferred the patient from the bed to the parent for KC.

The quantitative data collection was conducted over a 90 min period and comprised three phases, each lasting 30 min. This duration was specifically selected to facilitate optimal EEG assessment while minimizing the physiological burden on preterm infants. In phase 1, quantitative data from infants was collected during KC alone (baseline 1). In phase 2, the same data were collected during KC with music therapy intervention. Phase 3 was conducted after finishing music therapy, again with KC alone.

During and after the 90 min of data collection, the music therapist ensured the well-being of the parents and premature babies. Afterward, the nurse transferred the premature babies back to their beds, and the EEG electrodes were removed.

#### 2.4.2. Qualitative Data

The qualitative data were generated from semi-structured guided interviews with the parents of preterm infants by two NICU nurses [20]. The first author created interview guidelines. To devise the interview questions, the existing literature was reviewed and discussed in a tutorial led by the third author at IMC FH Krems, University of Applied Sciences. Before the interviews were conducted, a trial run took place with a fellow student and the first author to evaluate the interview guide. After discussing certain qualitative interviewing techniques, the first author and the two NICU nurses conducted the interviews using the guidelines in compliance with the data protection guidelines. The interviews lasted a maximum of one hour per parent. The guideline served exclusively as an aid for the study staff to collect information on various topics. The sequence of the questions could be flexibly adapted during the interview (Table A1).

### 2.5. Intervention

Each music therapy intervention during KC lasted approximately 30 min and consisted of 15 min of music and 15 min of introductory and concluding conversations between the music therapist and the parents.

The parents were lying on comfortable nursing chairs, and their premature babies were lying in KC. The music therapist gathered information about the parents’ current states of health and musical preferences to adapt the music therapy to their individual needs. Afterward, the parents were helped to focus on their breathing.

An interactive music therapy aligned with their respiratory pattern, based on CMT [11,21], was then conducted. This music therapy, tailored to the parents’ breathing rhythms during KC and freely improvised, was conducted for all subjects for about 15 min. The sessions started with an initial humming accompanied by the harp (Bohemian harp). The harp is the principal instrument employed by the music therapist in this research project, chosen for its resonant properties and ability to produce soft, gentle sounds that have been previously associated with calming and stabilizing effects in both infants and their parents [22]. After a while, the voice faded out, and the harp music continued playing a simple, gentle melody with repetitive rhythms in C major. After 15 min, the harp music faded out, and the music therapy ended.

After the music was adapted to the individual therapy situation, the music therapist shared her observations and perceptions of the preterm infants’ reactions to the music. The aim was to strengthen the parents’ perceptions and to support the interaction between parents and children.

### 2.6. Statistical Quantitative Analysis

Descriptive statistics were used to analyze the quantitative data. The raw data were prepared accordingly to make it accessible for the evaluation procedure [20]. The raw data consisted of heart rate, oxygen saturation, and brain activity (EEG) measurements in preterm infants, collected at the bedside using a Philips IntelliVue X3/MX100 monitor (Philips Healthcare, Amsterdam, The Netherlands) and a Natus Neurology portable video EEG device. Oxygen saturation and heart rate data were recorded every 5 min, transferred to Excel as trend charts, and used to calculate averages. Using diagrams and a detailed description, it was possible to show the course of the values over the three phases of data collection.

The EEG measurements were recorded in combination with video footage and then evaluated and written down by the principal investigator and the second author. First, the EEG and video recordings were medically analyzed by the second author and interpreted according to the Atlas of Neonatal Electroencephalography [23]. The second part of the analysis was conducted with the principal investigator and included the notes she made after each music therapy session. These notes included incidents and observations that might not have been visible on the video but were important for the analysis (noise, disturbances, etc.). Afterward, the EEG recordings were analyzed together a third time, and salient facts were noted per subject. Depending on the preterm infant’s gestational age, the expected characteristics were first defined, and the data of the feasibility project subjects were then collected. In addition to this qualitative description, the duration of each interburst interval (IBI) was measured in seconds, and the 3 IBI-maxima before and after initiation of music therapy were summarized for each subject. IBIs are quiescent periods of cerebral activity between bursts of high activity.

The difficulty in interpreting EEGs in preterm infants stems from constantly shifting typical patterns. The maturation of brain function is characterized by changes in continuous and discontinuous patterns during quiet and active sleep and wakefulness. Furthermore, there are typical patterns in transient sleep, in particular, beta–delta complexes and frontal sharp wave transients [23]. A discontinuous pattern is characterized by short bursts of high activity divided by IBIs. IBIs have characteristic durations depending on gestational age [24]. Prolongation of IBIs may be induced by sedation [25].

### 2.7. Qualitative Analysis

According to Glaser and Strauss, grounded theory-oriented coding was used [19]. Grounded theory implies the formation of theories from a network of concepts and enables a description and explanation of social phenomena [19].

The qualitative data analysis was conducted in an ongoing process that extended from the early stages of data collection through the data analysis to this study’s reporting. First, the first author listened to the audio recordings of the interviews and transcribed them in several passes. During transcription, short memos on key insights and observations were created and shared with the study team. After transcription, the interviews were analyzed using Atlas.ti (version 8.0). The first author marked relevant sections and coded them using open coding [19]. The codes were then discussed with the supervisors and organized into thematic code groups. Axial coding was then performed to draw connections between the code groups, refine and differentiate existing concepts, and form categories [19]. Finally, two major categories were formulated, which were used to answer the research questions.

## 3. Results

### 3.1. Demographic Data and Study Feasibility

All four parents who were asked to participate in this study gave their informed consent for study participation, adhered to the study protocol, and the whole study procedure could be conducted as planned. Parents even reported that they were interested in this study and satisfied with the study implementation, as outlined in more detail in the qualitative result section.

Six preterm infants between 29 and 34 weeks of gestational age were selected (May–July 2020) and included in the study period (Table 1). Three female and three male subjects were chosen, all delivered by Cesarean section (see Table 1). Among these subjects were two pairs of twins and two singletons. Two infants, subjects 1 and 2, were provided with non-invasive respiratory support (high-flow nasal cannula) during the quantitative data collection. Quantitative data collection was conducted during KC with the mothers and in one patient with the father (Table 2). The mother tongues of the participating parents were German (in two cases), English (in one case), and German and Albanian (in one case). The age range of the parents was 22–33 years.

### 3.2. Results of the Quantitative Analysis

Results of the Oxygen and Heart Rate Measurements

**Figure 1:**
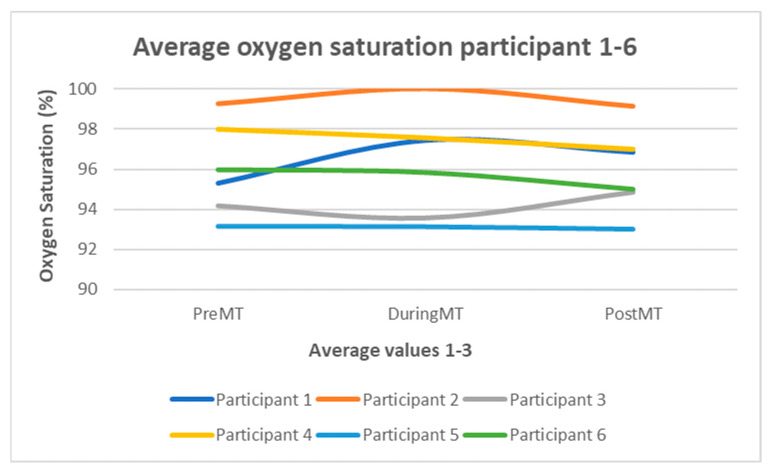
Average oxygen saturation subjects 1–6.

Figure 1 shows the mean oxygen saturation (SaO_2_) values of all six subjects during the three phases of data collection. In summary, the SaO_2_ mean values of all subjects in phase 1 ranged between 93 and 99%, phase 2 between 93 and 100%, and phase 3 between 93 and 99%. Thus, no significant fluctuations between the three phases were evident. Only in subject 1 was a minimal tendency of increase in SaO_2_ between phases 1 and 2 detected.

**Figure 2:**
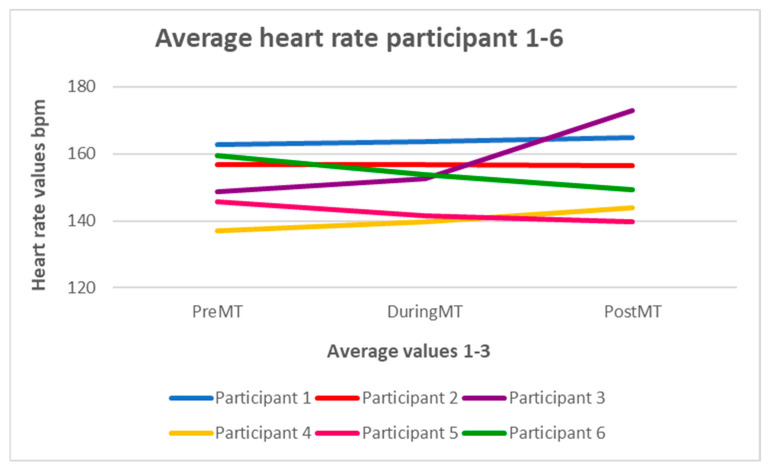
Average heart rate for subjects 1–6.

Figure 2 shows the mean values of the heart rate (hr) of all six subjects during the three phases of data collection. In summary, every subject’s mean heart rate values in phase 1 moved within a frequency range of 137–163 bpm, in phase 2 between 140 and 164 bpm, and in phase 3 between 140 and 173 bpm. The heart rate showed no significant change either. There was a minimal tendency of increase from phase 1 until phase 3. In subject 3, there was an increase in heart rate during the transition from phase 2 to 3, and in subject 6, there was a steady reduction in heart rate from phase 1 to 3.

### 3.3. Results of the EEG Interpretation

During initial EEG analysis, changes in the duration of the interburst intervals after initiation of music therapy were observed. Considering this finding, the interburst intervals were measured in seconds, and the duration before and after initiation of music therapy was recorded. Finally, the three maxima in each phase were summarized (Table 3). Based on these datasets, a tendency for prolongation of the interburst intervals after initiation of MT was noted compared to interburst interval durations before initiation (Table 3). This was the case in P2, P3, P4, P5, and P6. In P1, no prolongation of the IBI duration was seen because the EEG pattern fluctuated between discontinuous and continuous. This was interpreted as a change between active and quiet sleep.

**Figure 3:**
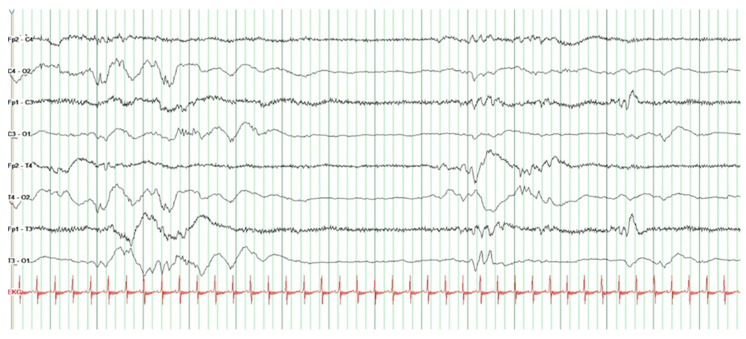
Discontinuous pattern (short version) 30 + 4 GW subject 2.

**Figure 4:**
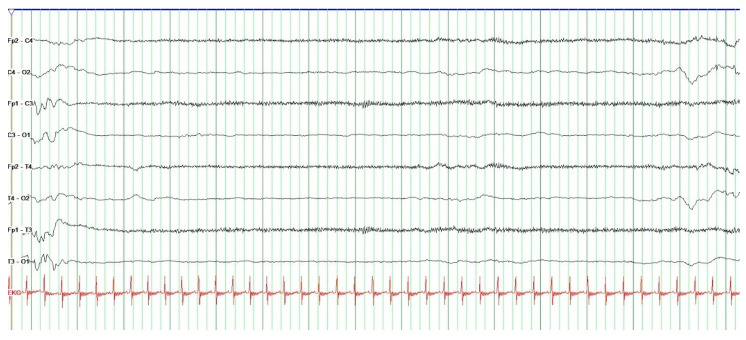
Discontinuous pattern (long version) 30 + 4 GW subject 2.

A short and long interburst interval in subject 2 is shown to illustrate the difference between the two (Figure 3 and Figure 4). Data collection was performed for subject 2 at 30 + 4 GW on postpartum day 11.

### 3.4. Results of the Qualitative Analysis

**Figure 5:**
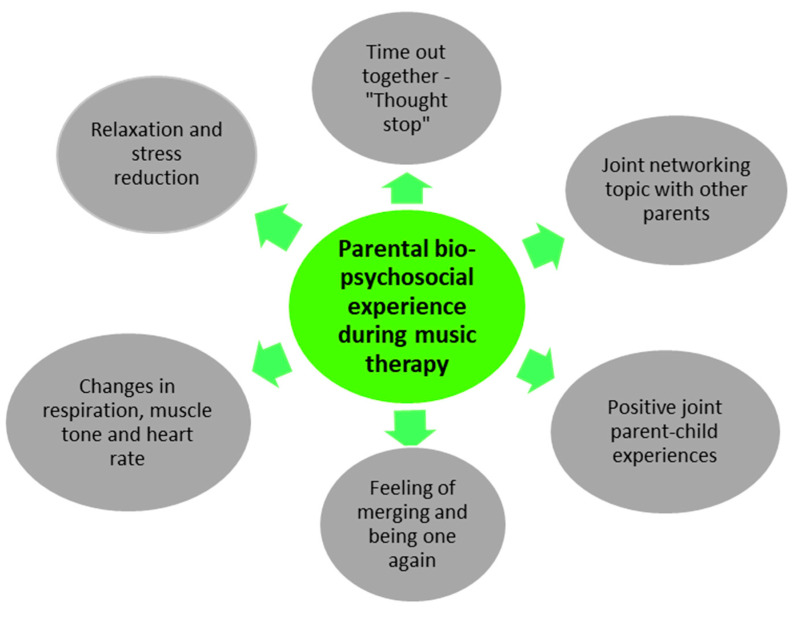
Main category 1: Parental experience on a biopsychosocial level during music therapy.

**Figure 6:**
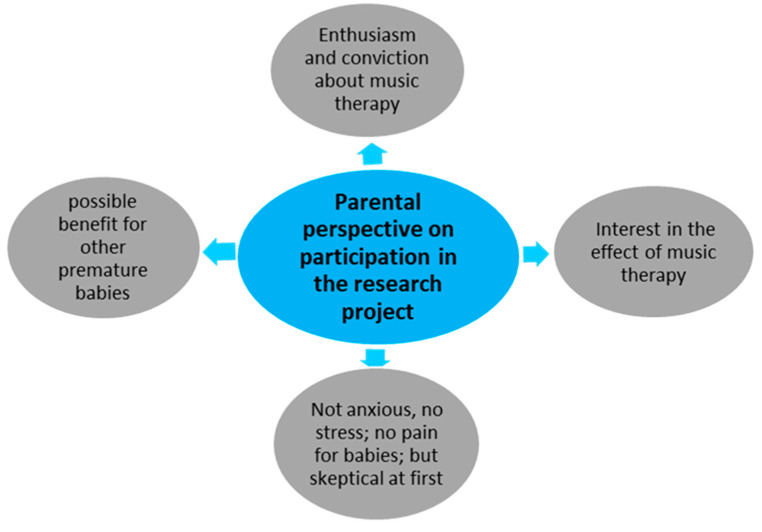
Main category 2: Parents’ perspectives on participation in the research project.

Two main categories (Figure 5 and Figure 6) were formed from the 11 code groups when analyzing the qualitative data. The first main category focuses on the *parental experience during music therapy on a biopsychosocial level* (Figure 5). The second main category contains those code groups that summarize the *parents’ perspectives on participating in this feasibility project* (Figure 6). Three code groups contain codes irrelevant to answering the research questions. Consequently, they have not been assigned to any main category.

Main category 1 consists of the following six code groups, as shown in Table 4, with the number of codes in brackets.

Main category 2 consists of two code groups, as shown in Table 5, with the number of codes in brackets.

Main category 1: “Parents’ experiences on a biopsychosocial level during music therapy

Main category 1 consists of six code groups and a total of 229 codes that shed light on the parents’ experiences at different levels during music therapy.

Parental experience of psychological parameters through music therapy

The largest code group, “Parental experience of psychological parameters through music therapy”, contains 94 codes and describes the parents’ perceptions of psychological parameters in themselves and their children during music therapy.

*“In the beginning, especially when I was an inpatient, it was difficult with all the equipment of an intensive care unit and the intensive care noise and the stress it caused. Music therapy was liberating and relaxing. It was great.”*

(Nora 4, 15)

The effect of relaxation and stress reduction on parents was a predominant topic in the interviews. The interviews revealed that music therapy was a positive, calming experience and reduced the stress in a way they had not experienced before during KC without music therapy. Some parents mentioned a feeling of deeper relaxation in themselves and their children; some even fell asleep during music therapy. In addition, some parents described music therapy as a “time-out” from their current situation, a thought stop, and a kind of shift in focus. An improvement in the parents’ perceptions of their children, such as relaxation signs during music therapy, was also mentioned several times. The following adverbs of “finally”, “suddenly”, and “for once” indicate that music therapy facilitated the relaxation they waited a long time for and describe the exceptional compared to the norm, e.g., KC without music therapy.

*“I could also **finally** relax, and my child and I calm down a bit. The thoughts could rest **for once**”*.

(Maureen 2, 15)

*“**Suddenly** there were these positive images in my head while the music was playing, and it was just amazing and completely fascinating”.*

(Nora 4, 10)

*Parental experience of physiological changes through music therapy*

In this code group, 45 codes were used to describe changes in respiration, muscle tone, and the general condition of premature infants and their parents in the context of music therapy. Several times, parents noticed a calmer and more regular breathing pattern in their children during or sometimes even after music therapy. Some parents also observed that their own breathing patterns were self-regulated positively. Some mentioned that the children became calmer overall, and sometimes, a slower heartbeat was noticeable compared to “normal” KC time.

*“But then towards the end, I got very tired, and I saw how he let everything hang and relaxed. **Normally** he always moves a lot, but at the end, he just lay on top of me completely relaxed and didn’t move at all.”*

(James 3, 42)

*Music therapy and parent–infant bonding*

This code group describes changes in parent–child bonding in the context of music therapy using 30 codes. The feeling of being one again and experiencing a familiar togetherness was mentioned several times. In summary, the main themes of this code group were the emergence of positive shared experiences for the family; the familiar, relaxed togetherness when cuddling; and the feeling of merging in the context of music therapy.

*“Yes, it was just positive experiences for the three of us together, and cuddling with the children gives me happiness. It was just great with the music, and I can only recommend it to everyone.”*

(Mia 1, 18)

*Special moments from the parents’ points of view (17)*

As special moments, this code group of 17 codes summarizes the main themes of the feeling of merging and being one again, the emergence of positive images of the future, and the sharing of beautiful experiences in the context of music therapy. The mother’s feelings about having her child in her womb again:

*“**A special moment**, yes, as already mentioned, how I had the feeling, is she there now or in my belly again, that was already a strange feeling—a kind of merging, you don’t know that either.”*

(Mia 1, 22)

*Emotional–social experience for parents through music therapy (29)*

In this code group, 29 codes are used to describe the emotional–social experiences that the parents had during music therapy. It was mentioned several times that parents enjoy music therapy with their children and that kangarooing together during music therapy was experienced as a positive, pleasant, and recommendable experience for the family. In addition, the therapeutic conversations during music therapy were described as calming, and music therapy was sometimes mentioned as a common way to network with other parents.

*“You get to calm down, even if it’s just for such a short time, **for once** you don’t listen to the beeping and the environment. I could just calm down and enjoy cuddling with my child and we talked a lot also about me and she explained some things to me. That was reassuring, and this is what other parents told me too.”*

(Maureen 2, 11)

*Previous music therapy experiences (14)*

This code group clearly shows, with 14 codes, that most of the parents had no previous knowledge or experience of music therapy before coming to the clinic.

*“No, I have never heard or known about music therapy before, but I like it a lot”.*

(Mia 1, 8)

*Main category 2: “Parents’ perspectives on participation in the research project”*

This main category contains the two code groups, “Feedback from parents on quantitative data collection” and “Reason for participation in the study”, with a total of 96 codes.

*Feedback from parents on quantitative data collection (50)*

Through this code group, it became apparent through 50 codes that the placement of the EEG electrodes in premature babies was perceived by their parents as not frightening or irritating, problem-free, not stressful, or painful, but it took some time to get used to.

The additional dressing applied by the EEG team helped the parents as it made it look as if their children had a bonnet on. In a few codes, it became clear that some parents were skeptical of this study. It was mentioned particularly often that the information provided by the music therapist in advance was helpful and that there were no suggestions for improvement regarding implementing this study and music therapy.

*“Yes, not scary but you have to get used to it, let’s put it that way. It takes some getting used to, but it’s not so bad. When we put the electrodes on, they were completely relaxed, they wouldn’t have protested at all. I don’t think they noticed it at all.”*

(Int1, 25)

*Reason for participation in the feasibility project (19)*

The parents’ reasons for participating in the pilot project are summarized in this code group based on 19 codes. The main reasons were **interest in the effects of music therapy** and the observation of the **process of a study**. **Enthusiasm and conviction** about music therapy and the **possible benefit for other premature babies** were also given as reasons for participation.

*“I just wanted to try it out. I was kind of interested in how it works and how such a study works. I just wanted to try it out. I wanted to know how it is and works for the babies as well and I thought I’ll just try it out now, why not?”*

(James 3, 32)

*Integration of quantitative and qualitative findings*

By analyzing the quantitative measurements of heart rate and oxygen saturation, in summary, vital signs in all subjects were stable throughout all study phases without any negative effects. In the qualitative data of this research project, parents repeatedly mentioned positive changes in the heartbeat and breathing of their premature babies in connection with music therapy. This was also mentioned in connection with their level of relaxation and the reduction of their stress and anxiety levels.

In addition, the medical evaluation of the EEG measurements based on the video recordings also clearly showed that the parents’ well-being and reactions influence the children. It was observed several times that the calmer and more relaxed parents were during data collection, the more pronounced calmer phases were in the EEG of the premature babies. The qualitative results again confirm this, with parents mentioning their own calming and a reduction of their heart and respiratory rate and muscle tone several times. Interestingly, the quantitative results, although statistically not significant, showed a slight increase in oxygen saturation in subjects 1 and 2 compared to subjects 3 and 4, who showed a slight decrease in oxygen saturation and a minimal increase in heart rate during/after music therapy. However, considering the qualitative results, it appears that all parents of these subjects noticed a relaxation in themselves and were thus able to enjoy the music therapy and the shared KC better and block out the intensive care noise for a moment. In addition, clear signs of relaxation, calmer breathing, and improved perception of their children were also mentioned. Only one parent (participant 3) could perceive signs of relaxation in himself and the child, as well as a more regular breathing pattern of the child, but no slowing down of breathing. Moreover, an increase in the parents’ well-being and an improved perception in connection with music therapy were mentioned frequently.

## 4. Discussion

Premature birth is a drastic event that confronts parents and their children with numerous challenges and can cause anxiety and stress for the whole family.

This feasibility study, therefore, evaluated possible beneficial influences of music therapy on oxygen saturation, heart rate, and brain activity using EEG measurements in infants before, during, and after music therapy. The parents’ perspectives on this study and the music therapy were also assessed. Additionally, this feasibility study aimed to prepare for implementing further and larger projects to advance developmental support through music therapy.

### 4.1. Influence of Music Therapy on Physiological Parameters of Premature Babies

The descriptive analysis of the quantitative results of this feasibility project showed overall stable physiological parameters without negative deteriorations (oxygen saturation, heart rate, brain activity) in the subjects (preterm infants) during and after music therapy.

Furthermore, the progression curve in the diagrams showed that the oxygen saturation values remained stable throughout the data collection without significant fluctuations. This stabilization and improvement of oxygen saturation during and after music therapy is in line with the results of several studies, as reported in a systematic integrative review [21] and a meta-analysis by Standley [26]. In contrast, two current meta-analyses by Bieleninik et al. [14] and Yue et al. [15] show no overall significant beneficial effect of music therapy on oxygen saturation in preterm infants. The different results presented in the latter two meta-analyses may be attributable to the heterogeneity of the studies examined or inclusion factors different from those chosen for the systematic integrative review [21] and the meta-analysis by Standley [26]. Our group of participating preterms had oxygen saturation levels within the normal range right from the beginning, leaving little room for improvement. Moreover, fluctuations in oxygen saturation during sleep are considered physiological in neonates. Even though it seems that most participants were more relaxed and had a proposed, deeper sleep, oxygen saturation stayed stable in the normal range.

The mean heart rate values of all subjects across the three phases of data collection changed minimally and did not improve substantially. This is in line with the meta-analysis results of Bieleninik et al. [14], who demonstrated a beneficial trend of music therapy on heart rate but no significant effect. Schlez’s randomized controlled trial [22] also confirms that there is no apparent effect of music therapy combined with KC on oxygen saturation and heart rate compared to KC alone. In contrast, the meta-analysis by Yue et al. [15] shows a beneficial effect of music therapy on heart rate and respiratory rate, oral feeding volume, stress level, and maternal anxiety in its examination of 13 studies with 1093 subjects. A methodological difference was that the voice of the music therapist was not used in this analysis. This may explain the lack of obvious effects on the physiological responses of the infants tested in Schlez’s study [22].

It is known that premature infants are particularly responsive to their mother’s voice. A female voice may, therefore, help promote relaxation and regulate physiological parameters. In our feasibility project, the voice of the female music therapist was only used at the beginning in the form of humming alongside the harp music. Stabilization or improvement of vital parameters could be achieved by using the voice for longer. In comparison, a randomized controlled trial [27] showed, among other things, that the music therapy intervention led to improved mental health in the mothers and a positive effect on the emotional arousal of the preterm infants as well as positive developments in reducing heart rate, stabilizing oxygen levels, and enabling earlier discharge.

Another reason for the different results for oxygen saturation and minimal changes in the heart rate may be that phase 1 of the data collection took place early, during KC. This activity has already been shown to stabilize the vital signs of premature infants [28]. The oxygen saturation recorded in these infants before music therapy was already in the upper range; consequently, the possible scope for the increase was limited.

This is in line with the results of the randomized controlled trial by Schlez et al. [22], which showed no apparent effect of music therapy combined with KC on oxygen saturation and heart rate compared to KC alone. Interesting information is provided by a crossover study by Span et al. [29], in which a combination of live music therapy and KC was just as beneficial for the physiological stability and neurological function of premature babies as live music therapy alone. In contrast, our participants showed a stabilization/improvement of the vital parameters in some subjects through KC and in others only through music therapy.

Data from EEGs and videos recorded during the intervention were analyzed to identify and contextualize any alterations in the subjects’ electroencephalographic patterns across the different stages of the intervention. The results may be influenced by age-related variations in patterns and waves. Notably, the EEG analysis revealed a tendency for interburst intervals (IBIs) in the discontinuous phases (deep sleep phases) to lengthen during and/or after the music intervention compared to baseline. To our knowledge, there are currently no publications addressing this finding. It is well-established that, for example, sedatives can prolong IBIs [25]. An extension of the IB intervals may indicate cerebral dysfunction, which, however, cannot be attributed to a response to music therapy. The most plausible explanation for this observation is a deepening of sleep, similar to the effects induced by sedative agents. However, supporting evidence for this hypothesis is currently lacking. The clinical presentation at this stage, along with the occurrence of IB intervals during the resting phase, suggests a deepening of sleep. We therefore hypothesize that the prolongation of IBIs in these subjects correlates with an increasing depth of sleep. It is further postulated that this may benefit the development of preterm infants. Further investigation of this observation using aEEG/CFM studies with a focus on IBIs could be valuable. Proving a positive effect on the neurological development of preterm infants was not the objective of this project. However, the results suggest such a connection.

These results are in line with the RCT by Haslbeck et al. [8], in which significant effects on the functional brain activity and brain development of premature infants using CMT were proven for the first time. In addition, a randomized controlled research project by Giordano et al. [12] confirms the positive effect of music on amplitude-integrated EEG (aEEG) activity in preterm infants. An aEEG study by Olischar et al. [13] also confirms that music may have a small effect on quiet sleep in newborns. A review article by Chorna et al. [30] analyzed fetal and neonatal processing of music and confirms our statement that further clinical study is needed to investigate the effects of different musical experiences and interventions on the development of preterm infants.

### 4.2. Parents’ Experiences and Perceptions of Music Therapy Research

The qualitative results show that music therapy creates a setting in which parents and their children can enjoy time together, relax, and, for a moment, block out the worries and the background noise of the intensive care unit, also compared to KC alone. Various mixed-methods pilot projects [17,31] corroborate the results of this small-scale study, which align with the reported relaxing and stabilizing effects for parents and children. In the study by Arnon et al. [32], a significant beneficial alteration in heart rate variability was observed during KC with maternal singing throughout both the intervention and recovery phases, compared to KC alone and baseline, and in the study of [22], maternal anxiety decreased more in the study arm with combined music therapy and KC than with KC alone. In addition, the meta-analysis by Bieleninik et al. [14] and the randomized controlled study by Schlez et al. [22] described reduced stress and maternal anxiety through music therapy.

On several occasions, the parents observed a slowing down of their own and their children’s breathing patterns during music therapy. They also noticed positive changes in the muscle tone of the premature babies. Parents were thus able to observe a relaxing effect on themselves and their children and, in some cases, a slowing down of the child’s heartbeat.

Similar results were shown in a randomized controlled pilot trial [31] in which family-centered music therapy also provided relief and relaxation for parents and premature infants, as well as promoting parent–child interaction and, subsequently, the development of a stable bond. Similar results were shown in a mixed-methods study [17], where a feeling of connectedness through musical interaction between parents and their children was described.

In contrast, a randomized controlled trial conducted in eight different NICUs in five different countries shows no significant differences between the music therapy and control groups in terms of mother–infant attachment, parental anxiety, or maternal depression [33]. The project [33] investigated the impact of a music therapy approach on mother–infant attachment and the mental health of parents of preterm infants, in which parents sang to their child under guidance during their time in the NICU. The results of this study differ greatly from those of the numerous studies cited above, and clarification is needed on whether this is due to the different forms of music therapy applied, the possibly shorter intervention length, or even the assessment tools. An interesting point of discussion is the timing of, for example, the assessment of mothers’ anxiety levels. In Gaden et al. [33], these were assessed just before discharge when they may be at their highest as parents are about to take full responsibility for their child at home. Interestingly, the results of Kehl et al. [34] showed significant levels of anxiety reduction two weeks after birth and halfway through the hospital stay, but this was no longer apparent two weeks after discharge. The reduction of maternal anxiety through music therapy has already been proven by numerous studies [14,22,32,34]. In addition, the positive effects of music therapy on parents’ well-being and parent–child bonding have also been confirmed several times [11,17,34,35,36,37].

The most common reason for parents to participate in our research project was their interest in the effect of music therapy and how a study in this field was conducted. The results show that data collection did not trigger anxiety or stress in parents. In summary, the parents were able to perceive positive effects for themselves and their children during music therapy sessions and during the research project. This feasibility project showed that integrating parents into the research process is of great importance, for example, by integrating their perspectives into the design and conduct of studies and thereby addressing the needs of parents and children. A study by Janvier et al. [38] looked at the integration of parents in neonatal and pediatric research and confirmed the indispensability of involving parents and children in clinical research in the future to explore important outcomes for parents and children. On the other hand, care must be taken not to overwhelm parents or trigger additional stress by involving them in research. The literature review by Shen et al. [39] draws attention to the current lack of a solid evidence base on how to effectively integrate parents as co-researchers.

## 5. Limitations

Limiting factors of the current study are the small number of participants, a lack of statistical significance, and the limited time frame. However, since the research team was already aware of this when planning the project, the objective was to descriptively report observation and feasibility as a starting point for future investigations.

To obtain better comparative values, the inclusion of premature infants of the same gestational age should be forced in follow-up studies.

Critically reflected, a higher number of test persons, randomization, and frequent repetition of the quantitative measurements would be crucial for a follow-up study. Furthermore, randomization into a group with music therapy during KC and a control group without music therapy is needed. In a follow-up project, using an amplitude-integrated EEG (aEEG) focusing on the interburst intervals would be helpful in further explaining the observations made. In the EEG analysis, it would be worth considering determining the entire course of the interburst intervals and calculating their minimum, maximum, and mean values.

For an optimized survey of parental perception and well-being, follow-up projects could, for example, measure the stress level through self-assessment using rating scales. Randomized controlled trials in hospitals must be conducted to further deepen the new findings and advance the developmental support of preterm infants.

In addition, research projects that investigate music therapy’s long-term effects on preterm infant development should be funded.

## 6. Conclusions

The results of this mixed-methods feasibility project indicate a possible positive influence of music therapy on physiological parameters and the brain activity (EEG) of premature babies. However, the results should be interpreted cautiously due to the small number of subjects.

Within the framework of this research project, it proved possible to determine a prolongation of the interburst intervals in the EEG, stable vital signs of premature babies in connection with music therapy, and beneficial factors for well-being and bonding between parents and their premature babies. Our results indicate the usefulness of considering parents and children as a unit in the field of neonatology, not only in clinical practice but also for research purposes. The implementation of studies in the field of neonatology should be intensified, and a greater focus should be placed on the needs of parents and children as highly vulnerable individuals. Furthermore, such studies must meet high ethical standards to advance developmental support and ensure the best possible care for preterm infants and their parents.

## Figures and Tables

**Table 1:** Sociodemographic characteristics of subjects at baseline (premature babies).

Baseline Characteristics	*n*	%
Gender of the children		
Female	3	50
Male	3	50
Mode of birth		
Cesarean section	6	100
Spontaneous birth	0	0
Week of pregnancy at birth		
29 + 0	2	33
31 + 5	3	50
34 + 1	1	17
Singleton/multiple		
Single children	2	33
Twins	4	67
Respiratory support during data collection		
High-flow nasal cannula, 21% oxygen (O_2_)	2	33
Without respiratory support	4	67

**Table 2:** Sociodemographic characteristics of subjects at baseline (parents).

Baseline Characteristics	*n*	%
Gender of the parent		
Female	3	75
Male	1	25
Parents’ ages		
22	1	25
26	1	25
28	1	25
33	1	25
Native language		
German	2	50
German and Albanian	1	25
English	1	25
Professions of parents		
Retail sales assistant	1	25
Market manager	1	25
Board of director’s assistant	1	25
Production employee	1	25

**Table 3:** Results of the EEG measurements.

Subject	P1	P2	P3	P4	P5	P6
Gestational week (GW)	30 + 4	30 + 4	33 + 5	32 + 6	35 + 0	32 + 6
Reference values of average IBI duration in this GW	9 s–10 s (max. 20 s)	9 s–10 s (max. 20 s)	4 s–5 s (max. 20 s)	6 s (max. 20 s)	4 s (max. 10 s)	6 s (max. 20 s)
Changes in EEG pattern after the beginning of music therapy	Discontinuous to the continuous pattern, return to the discontinuous pattern	Prolongation of IBI	Prolongation of IBI	Discontinuous to the continuous pattern, prolongation of IBI after MT	Prolongation of IBI, recurrent fluctuation of continuous/discont. pattern	Initially discontinuous to continuous, then prolongation of IBI
Three maximum IBI durations before music therapy	14 s, 16 s, 19 s	6 s, 7 s, 14 s	3 s, 4 s, 6 s	13 s, 17 s, 17 s	9 s, 12 s, 12 s	11 s, 12 s, 13 s
Three maximum IBI durations after beginning music therapy	12 s, 13 s, 16 s	15 s, 22 s, 19 s	10 s, 12 s, 15 s	18 s, 21 s, 21 s	13 s, 14 s, 14 s	16 s, 18 s, 20 s
Disturbing factors, as seen in the video and EEG	Restlessness, hiccups	Muscle artefacts	Arousal reaction from infusion alarm	Muscle artifacts; wakefulness phase	Restlessness	Movements

**Table 4:** Category 1: Parents’ experiences on a biopsychosocial level during music therapy.

Parental experience of psychological parameters through music therapy (94)
Parental experience of physiological changes through music therapy (45)
Music therapy and parent–child bonding (30)
Special moments from the parents’ points of view (17)
Emotional–social experience for parents through music therapy (29)
Previous music therapy experiences (14)

**Table 5:** Category 2: Parents’ perspectives on participation in the research project.

Feedback from parents on quantitative data collection (50)
Reason for participation in the study (19)

## Data Availability

The raw data supporting the conclusions of this article will be made available by the authors without undue reservation.

## Abbreviations

CMT	Creative Music Therapy
EEG	Electroencephalography
KC	Kangaroo Care
KUK	Kepler University Hospital
NICU	Neonatal Intensive Care Unit
NIMCU	Neonatal Intermediate Care

## Appendix A

Table A1 below provides an overview of the qualitative data collection, which took the form of four interviews. Column 1 shows the anonymized abbreviations of the six subjects, and column 2 shows the coding of the interviews. The abbreviations of the interviewers are shown in columns 3, and columns 5 and 6 show the codes contained per interview and in total (243 codes), with allocations to the 11 code groups.

**Table A1:** Interview constellation.

Personal Abbreviation	Interview Abbreviation	Abbreviation Interviewer	Codes Interview	Codes Total	Code Groups Total
P1 + P2	Int1	I1	32	243	11
P3	Int2	I1	30		
P5	Int3	I2	67		
P4 + P6	Int4	I2	45		
